# Effect of Interfacial Compatibility on Mechanical Property of Polyamide 6 Modified by Polyborosiloxane

**DOI:** 10.3390/polym17030392

**Published:** 2025-01-31

**Authors:** Qian Chen, Feng Li, Zhe Zhai, Shufeng Li, Yongfei Cai, Qiang Li

**Affiliations:** 1 School of Materials Science and Engineering, Xi’an University of Technology, No. 5 Jinhua South Road, Beilin District, Xi’an 710048, China; chenqian163cq@163.com (Q.C.);; 2 Quanzhou Peak Shoes Co., Ltd., Dongbao Industrial Zone, Fengze District, Quanzhou 362018, China; 3 China Helicopter Research and Development Institute, 602 Hangkong Road, Zhushan District, Jingdezhen 333001, China; 4 Jiangxi Lian Chuang Electroacoustic Co., Ltd., Yaohu West Sixth Road, Gaoxin District, Nanchang 330096, China

**Keywords:** silane coupling agent, polyborosiloxane, interfacial adhesion, interparticle distance, double-yield behavior

## Abstract

The interfacial properties of blends play a crucial role in determining the mechanical characteristics of polyamide alloys. This study focused on the preparation of PA6/PBS alloys via a melt blending method, utilizing 3-aminopropyltriethoxysilane (KH550) as the compatibilizer to examine the impact of KH550 on the interfacial and mechanical properties of these binary blends. The results demonstrated that the amino groups in KH550 reacted with the terminal carboxyl groups in polyamide 6 (PA6) and the B-OH in polyborosiloxane (PBS), which significantly enhanced interfacial adhesion between the two phases. A reduction in the particle size and interparticle distance of PBS particles was related to increased interfacial adhesion within the blends. The superior dispersion and robust interfacial adhesion caused a notable improvement in the notched Izod impact strength, rising from 7.9 kJ/m^2^ to 29.7 kJ/m^2^ at 25 °C and from 6.3 kJ/m^2^ to 16.6 kJ/m^2^ at −50 °C. Consequently, KH550 proved to be an effective toughening agent for the PA6/PBS system. Furthermore, the PA6/PBS blends containing a high content of KH550 induced a morphological transformation from a “sea-island” structure to a partially interpenetrating polymer network, leading to the absence of a double-yield phenomenon in the tensile curve.

## 1. Introduction

Polyamide 6 (PA6) is nowadays one of the most established engineering polymers with a wide range of applications in various engineering fields, including the automotive and aerospace industries, attributed to its high service temperature, good processability, and mechanical properties. PA6 is distinguished by a high concentration of amide groups (-CONH) along its main chain [1,2], which facilitates the formation of interchain hydrogen bonds, enhancing molecular cohesion compared to conventional thermoplastics such as polyolefins and polyesters [3,4,5]. Consequently, PA6 demonstrates remarkable thermomechanical properties along with exceptional fatigue and wear resistance [6]. However, the presence of amide groups with strong polarity in polyamide leads to low crack resistance, thereby resulting in weakened impact toughness, particularly under dry conditions and at low temperatures [7,8]. This limitation significantly restricts the application of PA6 in cold and dry environments.

To address this problem, toughening agents, including rubber elastomers [9,10], thermoplastic elastomers [11], metallocene polyolefin, core–shell copolymers [12], and rigid particles [13,14], are used to develop tough polyamide alloys via melt blending. The toughening ability of elastomer to the PA6 has been reported by several researchers [15,16]. According to Nielsen’s secondary transition temperature theory, the toughness of polymers is supposed to be linked to the secondary transition temperature. Silicone elastomers, known for their excellent low-temperature performance, present a viable option for enhancing the notch strength of PA6. A study conducted by Zhang et al. [17] demonstrated that blending polydimethylsiloxane (PDMS) with PA6, along with utilizing epoxidized silicone rubber as an “interfacial compatibilizer”, successfully improved the low-temperature toughness of PA6 from 2.3 kJ/m^2^ to 13.1 kJ/m^2^ at −60 °C. Polyborosiloxane (PBS) features a very low glass transition temperature, providing excellent resistance to brittleness at low temperatures. Thus, blending PA6 with PBS represents a promising strategy to improve its toughness in colder environments. Nevertheless, the significant difference in polarity between PA6 and PBS poses challenges concerning phase compatibility during blending, leading to microphase separation that affects the impact toughness of the polyamide alloys.

To improve the compatibility of the two-phase interface, a third component, such as a silane coupling agent [18,19], is typically added as a compatibilizer. Alternatively, a polar or reactive group, like maleic anhydride (MAH) or glycidyl methacrylate (GMA), can be introduced into one or both components to promote a more effective interaction between the two phases, thereby improving the binding strength of the interfacial layer [20,21]. Mirzaee et al. [22] reported that the impact strength of PA6 blends with the impact modifier styrene–butadiene rubber (SBR), using grafted glycidyl methacrylate, reached 539.8 J/m by adjusting the amounts and grafting degree of the compatibilizer. Additionally, Akira et al. [23] discovered that maleic-anhydride-modified styrene–ethylene–butylene–styrene copolymers (mSEBS) were controlled by adding paraffin oil and could act as an interface between PA6 and PP, resulting in an increase in the notched Izod impact strength of the ternary blend by over 60 kJ/m^2^. Therefore, regulating the structure and properties of the interfacial layer in blends is crucial for enhancing the toughness of polyamides [24].

KH550 contained a polar group that could theoretically react with the amide groups in the PA6 chain, affecting the interface properties of the binary blend. PA6/PBS blends with varying contents of KH550 are prepared in this study to investigate the effect of KH550 on the interface properties and mechanical characteristics. Based on the research, it is identified that the influence of the introduction of PBS and KH550 to PA6 would be a significant supplement to the field of elastomer toughening of PA6.

## 2. Experimental

### 2.1. Materials

PA6, identified by grade BL3280H, was supplied by Sinopec Baling. The material exhibited a melting temperature (Tm) of 220 °C and a melt flow index (230 °C, 2.16 kg) of 16.4 g/10 min. It is available in pellet form at a density of 1.12 g/cm^3^. Hydroxy-terminated polydimethylsiloxane (PDMS-OH), characterized by a hydroxyl content of 9.2%, was sourced from Tianjin Yongsheng Fine Chemical Co., Ltd. (Tianjin, China). Boric acid (H_3_BO_3_) was supplied by Tianjin Hengxing Chemical Reagent Manufacturing Co., Ltd. (Tianjin, China). Irganox 1098, a hindered phenolic antioxidant, was obtained from BASF (Ludwigshafen, Germany). Additionally, KH550, a silane coupling agent, was provided by Dongguan Kangjin New Material Technology Co., Ltd. (Dongguan, China).

### 2.2. Preparation of PA6/PBS Blends

PBS was synthesized from PDMS-OH and boric acid using the non-hydrolysis method. Initially, PDMS-OH was placed in a vacuum kneader, sealed, and stirred to 100 °C. Afterward, boric acid was added to the reactor, causing the temperature to rise to 250 °C, which was maintained for 30 min to ensure a complete reaction. Upon cooling, the PBS was obtained, with a molar ratio of silicon atoms to boron atoms of 4.75:1.

PA6/PBS blends were produced via melt blending and hot stretching. Prior to melting, PA6 and PBS were dried in a vacuum oven at 80 °C and 70 °C, respectively, for 24 h. Subsequently, the PA6, PBS, KH550, and antioxidant 1098 were combined in an internal mixer at 240 °C for 30 min. After cooling, the blends were processed using a crusher to break them down. Ultimately, the PA6/PBS-m masterbatches were prepared via extrusion melt blending in a twin-screw extruder, where m represented the content of KH550. The temperature profile across the eight heating zones during extrusion was set to 215 °C (feeding), 220 °C, 225 °C, 230 °C, 235 °C, 235 °C, 235 °C, 235 °C, and 235 °C (die). A feeding rate of 6 rpm was used, while the extruder screw speed was maintained at 260 rpm. All samples were subsequently dried in a vacuum oven at 100 °C for 16 h to remove any absorbed moisture before being injection molded at 250 °C. The compositions of the blends are presented in Table 1.

### 2.3. Characterization

#### 2.3.1. Fourier-Transform Infrared Spectroscopy (FTIR)

FTIR analysis was conducted using an Agilent Cary 630 infrared spectrometer with a spectral range of 4000 to 600 cm^−1^, taking a resolution of 8 cm^−1^ to analyze neat PA6 and PA6/PBS blends with different contents of the silane coupling agent. All samples were dried in a vacuum oven at 80 °C for 24 h prior to the measurements to remove absorbed moisture.

#### 2.3.2. Scanning Electron Microscope (SEM)

The blends were etched with isopropyl alcohol for 5 min to remove the PBS phase. Subsequently, the fractured surfaces of both neat PA6 and the PA6/PBS blends were sputtered with platinum to obtain a conductive surface prior to testing, preventing the effect of electrodeposition. The phase morphology was then observed using SEM at an accelerating voltage of 20.0 kV.

#### 2.3.3. X-Ray Diffraction (XRD)

The crystallinity and crystalline forms of the blends were determined using an X-ray diffractometer (Smart Lab, Japan) with Cu-Kα radiation (λ = 1.54 Å). Diffraction spectra were recorded in the 2θ range of 5–55° at a scan speed of 5°/min, with an accelerating voltage, current, scan speed, and step interval of 40 kV, 200 mA, and 0.01°, respectively.

#### 2.3.4. Differential Scanning Calorimetry (DSC)

The crystal transformation and glass transition temperature of the PA6/PBS blend were determined using a DSC 200 F3 Maia^®^ from NETZSCH (Selb, Germany) under atmospheric pressure of 0.03 MPa in a nitrogen atmosphere. To eliminate the thermal history, the PA6/PBS masterbatch was encapsulated into the sealed aluminum crucible and heated from 0 °C to 250 °C at a rate of 10 °C/min. Then, both heating and cooling procedures were conducted within the range of −150–250 °C at a rate of 5 °C/min. The melting enthalpy was provided by the second heating thermogram, and the crystallinity (*χ_c_*) of the blends was calculated using the following equation:

(1) $$\chi_{c}=\frac{{\Delta H}_{C}}{\Phi \Delta H_{0}}\times 100\%$$

where Δ*H_c_* is the melting enthalpy of samples, *Φ* is the mass fraction of PA6, and Δ*H*_0_ is theoretical enthalpy of melting for 100% crystalline PA6 (230 J/g) [25,26].

#### 2.3.5. Mechanical Testing

The notched Izod impact strength of the specimens in a dry state was evaluated at various temperatures using a pendulum impact testing machine (ZBC8000-B, MTS Systems Ltd., Sentosa, Singapore) with a 5.5 J hammer, following the standard GB/T 1843-2008 [27]. The dimensions for the 45 °V-shaped notch impact specimens were 80 mm × 10.0 mm × 4.0 mm (length × width × thickness), featuring a notch depth of 2 mm and a radius of 0.25 mm. Prior to testing, the specimens were conditioned at a temperature of 23 °C and relative humidity of 50% for 24 h. The average impact strength was calculated from 10 repetitions for each sample.

Tensile tests were conducted using a microcomputer control electronic universal testing machine (CMT4204, ShenZhen San Si, China) with a fixed crosshead speed of 50 mm/min, in accordance with GB/T1040-2018 [28]. The tensile strength and nominal tensile strain at fracture were reported as averages from 5 repetitions performed at room temperature. All specimens were dumbbell-shaped with a gauge length of 75 mm.

### 2.4. Therory and Model

The critical interparticle distance can be derived from the interparticle distance model proposed by Wu in 1985 [29], which describes the relationship between the critical particle diameter, critical interparticle distance, and volume fraction. The critical interparticle distance (*ID_c_*) is determined using Equation (2) below:

(2) $$ID_{c}=d_{wc}\left[{\left(\pi /6\phi_{r}\right)}^{1/3}-1\right]$$

where *ID_c_* signifies the distance between the surfaces of the two nearest neighboring particles. The parameters *d_wc_* and *ϕ_r_* represent the critical particle diameter and the volume fraction of the dispersed phase, respectively. The weight average diameter (*d_w_*) and volume fraction of the dispersed phase can be expressed as follows [30,31]:

(3) $$d_{w}=\sum n_{i}d_{i}^{2}/\sum n_{i}d_{i}$$

(4) $$\phi_{r}=\frac{\rho_{m}w_{f}}{\left[(\rho_{m-}{\rho_{f})w}_{f}+\rho_{f}\right]}$$

where *w_f_*, *ρ_f_*, and *ρ_m_* refer to the weight fraction of the dispersed phase, the density of the PBS, and the density of the PA6 matrix, respectively. For the PA6/PBS blends, the densities are given as *ρ_m_* = 1.13 g/cm^3^ and *ρ_f_* =1 g/cm^3^. Finally, by combining Equation (2), the interparticle distance (*ID*) values can be obtained.

## 3. Results and Discussion

### 3.1. Structural Characterization

**Figure 1:**
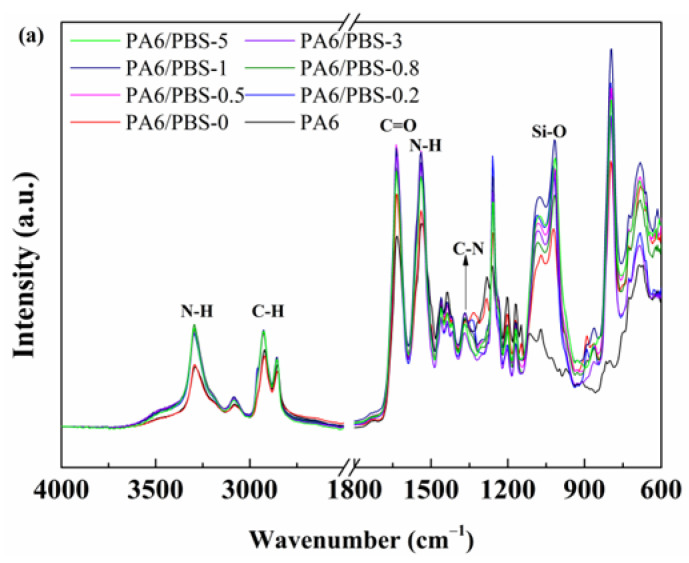

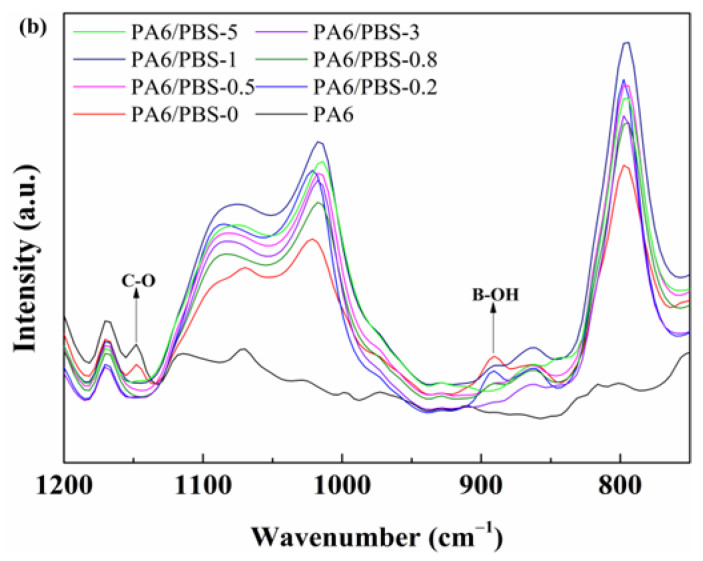
FTIR spectra of neat PA6 and the PA6/PBS blends: (**a**) full range; (**b**) amplified fingerprint area.

**Figure 2:**
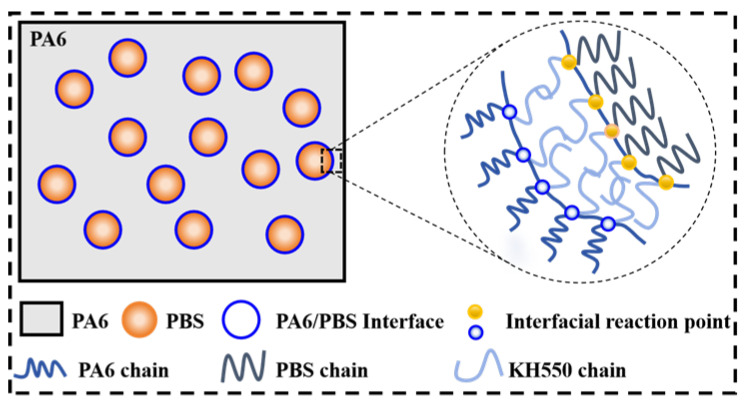
Diagram of PA6/PBS blends’ interfacial reaction mechanism.

It is crucial to assess whether a reaction occurred between KH550 and the PA6/PBS. Figure 1 plotted the FTIR spectra of neat PA6 and the PA6/PBS blends. Notably, PA6 exhibited an absorption peak at 3295 cm^−1^, indicative of N-H stretching vibrations, along with C-H symmetrical and asymmetrical stretching observed at 2851 cm^−1^ and 2922 cm^−1^, respectively. Furthermore, three characteristic peaks related to amide groups were present: C=O stretching vibrations (amide-I) at 1633 cm^−1^, N-H bending vibrations (amide-II) at 1532 cm^−1^, and C-N stretching vibrations (amide-III) at 1364 cm^−1^ [6,32]. It was anticipated that the incorporation of PBS would result in the appearance of characteristic peaks corresponding to Si-O stretching vibrations in the range of 1085–1018 cm^−1^ [33,34]. Moreover, the disappearance of C-O absorption peaks at 1148 cm^−1^ in PA6 upon the introduction of KH550 confirmed that a reaction occurred between the terminal carboxyl group in PA6 and the amino group in KH550. Additionally, the absorption peak at 891 cm^−1^, corresponding to the stretching vibration of B-OH in PBS, gradually faded as the content of KH550 increased. This observation demonstrated that the amino group in KH550 also reacted with the B-OH in PBS, which can be explained by the interfacial reaction mechanism illustrated schematically in Figure 2.

**Figure 3:**
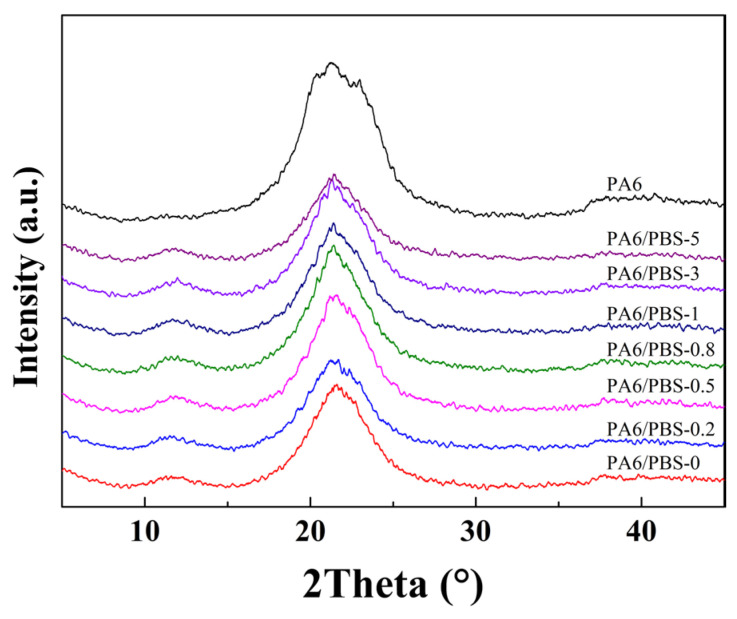
XRD spectra of neat PA6 and the PA6/PBS blends.

PA6 is a polycrystalline polymer, and distinct crystalline species can manifest under varying conditions, including α-phases with a monoclinic structure and γ-phases with a hexagonal structure. The characterization of these crystalline species in PA6-based composites can be achieved using XRD. As could be seen in Figure 3, three minor diffraction peaks were detected at approximately 2θ = 20.6°, 2θ = 22.9°, and 2θ = 21.5°, corresponding to the α(200), γ(002), and α(002/202) crystal planes of PA6, respectively. Notably, the γ-phases emerged as the dominant crystalline phase following the incorporation of amorphous PBS into the PA6 matrix. The γ-phases, characterized by parallel molecular chains, exhibit fewer intermolecular hydrogen bonds compared to the α-phases, which have a reverse parallel arrangement. This structure contributes to improved impact toughness in PA6. Furthermore, the amount of silane coupling agent had minimal effect on the crystal structure of the PA6/PBS blends.

### 3.2. Melting and Crystallization Behaviors

**Figure 4:**
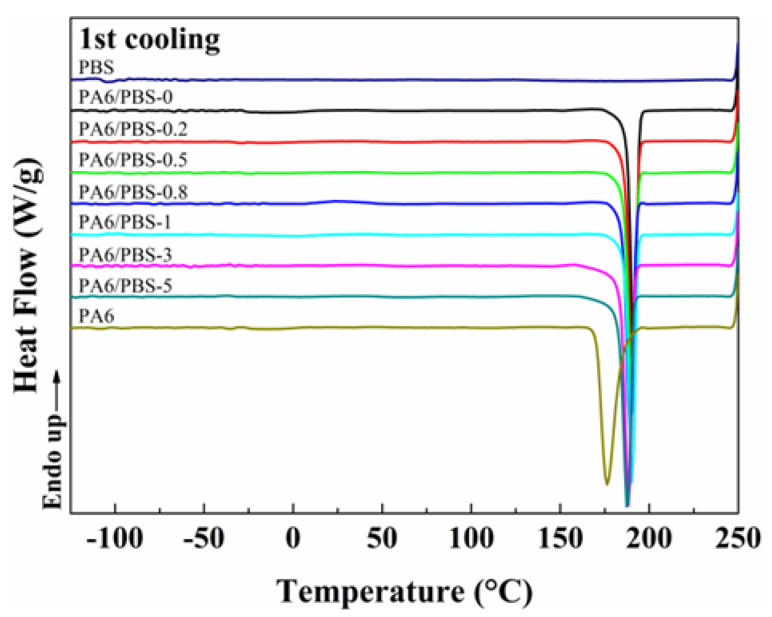

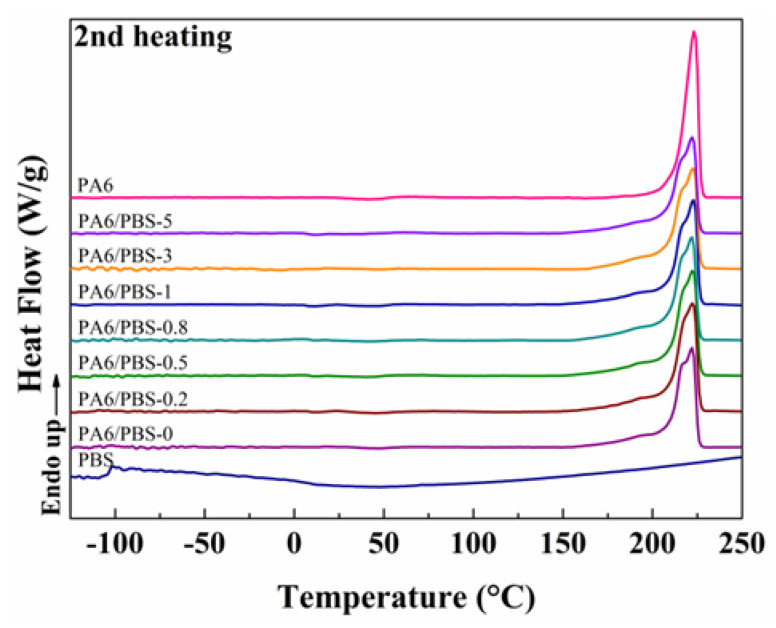
DSC first cooling (after 5 min isothermal hold for erasing heat history) and second heating curves of PA6 and the PA6/PBS blends.

**Figure A1:**
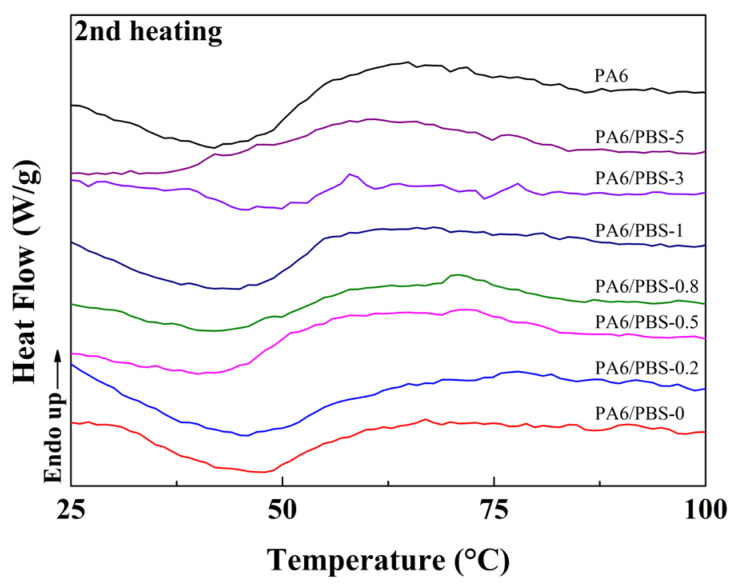
The amplified melting curves of PA6 and the PA6/PBS blends.

The mechanical properties of polymer blends relate to the crystallinity and crystallization behavior of the polymers. The effects of PBS elastomers and KH550 coupling agents on the melting and crystallization behaviors of PA6 and PA6/PBS blends were investigated through DSC analysis, as illustrated in Figure 4. It was observed that the α-crystals formed at approximately 222 °C, while the metastable γ-crystals appeared around 217 °C. The low-temperature peaks observed in the PA6/PBS blends can be attributed to variations in the crystal polymorphism of PA6; specifically, the addition of PBS induces a transformation of PA6 from α to γ-crystal. As the compatibility between the two phases improved, the initial crystallization temperature of the blends gradually decreased, while the type of crystallization and melting points remained unchanged. Additionally, the glass transition temperature of PBS was measured at −103.7 °C, whereas PA6 exhibited a glass transition temperature of 52.4 °C. Notably, increasing the KH550 content to 5 wt% results in a decrease of PA6’s glass transition temperature to 46 °C (the amplified melting curves can be found in Appendix A Figure A1). Consequently, incorporating PBS had the potential to enhance the low-temperature notched Izod impact strength of the blend, provided that the blend retained good compatibility.

Then, the crystallinity of PA6 and the binary blends was calculated using Formula (1), and the results are shown in Table 2. It was observed that the crystallinity of the blends decreased with the introduction of PBS, indicating that PBS inhibited the crystallization of PA6. Meanwhile, there was a significant increase in the crystallization temperature of the blends when the PBS phase was present since PBS functions as a heterogeneous nucleus in the PA6/PBS blends.

### 3.3. SEM Analysis

**Figure 5:**
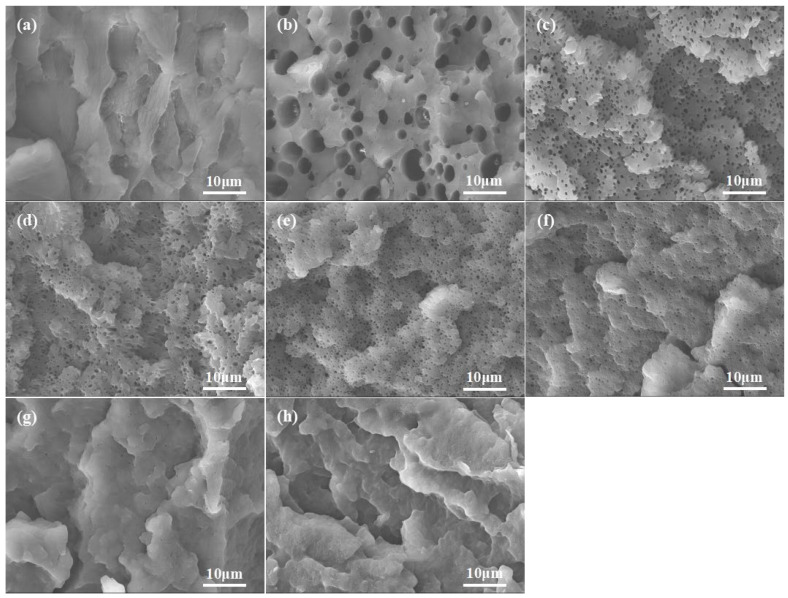
SEM images of PA6 and PA6/PBS blends: (**a**) PA6; (**b**) PA6/PBS-0; (**c**) PA6/PBS-0.2; (**d**) PA6/PBS-0.5; (**e**) PA6/PBS-0.8; (**f**) PA6/PBS-1; (**g**) PA6/PBS-3; (**h**) PA6/PBS-5.

In general, the mechanical properties of binary blends also depend significantly on the morphological structure of the blend, the size and distribution of the dispersed phase, interfacial adhesion, and interparticle distance. To investigate the morphology and distribution of the dispersed phase in PBS/PA6 blends with varying concentrations of KH550, isopropyl alcohol was used to etch the fractured surfaces. This process effectively removed the PBS phases, revealing numerous spherical pits on the fractured surfaces, as observed through scanning electron microscopy (SEM), with the results presented in Figure 5. It is evident that the surface morphology of the PA6/PBS blends exhibits a “sea-island” structure, where PBS acted as the dispersed phase and PA6 as the continuous phase.

The PBS particle sizes in the PA6/PBS blends without silane coupling agents were larger and unevenly distributed with well-defined boundaries. This observation suggested weak interface adhesion between the two phases, a consequence of the poor compatibility stemming from the significant polarity difference between PA6 and PBS. When the blended system was subjected to impact, the PBS particles triggered stress concentration within the matrix, resulting in the formation of crazing. Owing to the weak interfacial adhesion between the two phases, the PBS phase tended to debond during the impact process, allowing the crazing to grow rapidly without the constraints of interfacial adhesion, ultimately leading to the formation of cracks. As a result, the blend exhibited characteristics of brittle fracture.

With the inclusion of KH550, the fracture surface of the blends, characterized by irregular structures resembling “scales”, exhibited a rougher surface. The PBS phase was uniformly dispersed in the PA6 matrix, primarily due to the reaction between the amino groups in KH550 and the terminal carboxyl groups in PA6, as well as the B-OH in PBS. This interaction significantly enhanced the interfacial adhesion between the two phases. The interface adhesion surpassed the agglomeration force of PBS particles, which further restricted the aggregation of PBS particles. Consequently, with an increasing content of KH550, the interface adhesion of the blends progressively improved, leading to a gradual reduction in the particle size of PBS. Notably, when the content of KH550 in the blend reached 5 wt%, virtually no voids were observable in the fracture surface of the blends after isopropyl alcohol etching. This phenomenon primarily arose from the robust interfacial adhesion in the blend, the reduced particle size of PBS, and the expansion of PBS during the etching process. Additionally, the enhanced compatibility of the blend also contributed to the degree of diffusion between the two phases, resulting in a more indistinct blending interface and an increase in the thickness of the interface layer, thereby influencing the morphology of the blend.

**Figure 6:**
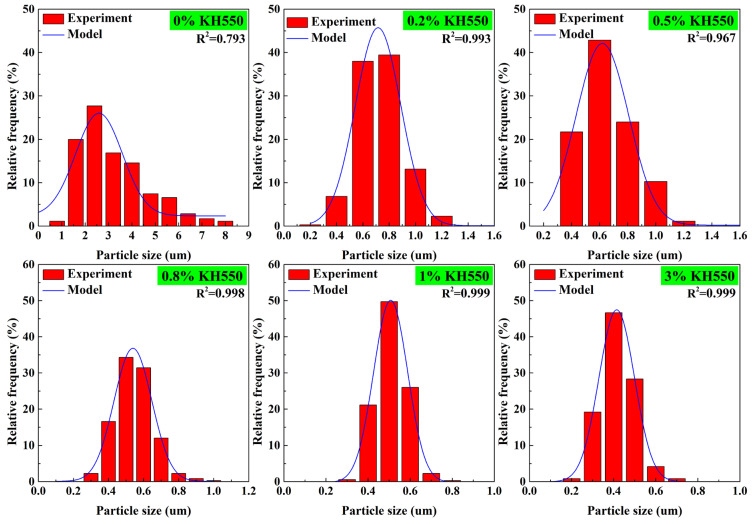
Histograms of PBS particle sizes and size distributions in PA6/PBS blends with different KH550 contents.

The particle sizes and size distributions of the PBS particles at various contents of KH550 were subsequently estimated using Nano Measurer1.2 software based on SEM images, as depicted in Figure 6. The particle size of PBS exhibited a Gaussian distribution, with R^2^ representing the correlation coefficient. A value of R^2^ approaching one indicated that the particle size distribution of the PBS aligns closely with a Gaussian distribution. As the content of KH550 increased, the R^2^ coefficient gradually rose, while the particle size range of PBS decreased, implying a commendable dispersion of blends. By referencing Figure 5 alongside Equations (2) and (3), the interparticle distance and weight average diameter of the PA6/PBS blends were calculated to evaluate the impact of KH550 on the brittle–ductile transition of these blends. The data presented in Table 3 revealed that the incremental increase in the content of KH550 from 0 wt% to 3 wt% resulted in a substantial reduction in both the interparticle distance and particle size. This effect could be attributed to the enhanced interfacial adhesion between the two phases on account of the reaction between the amino group in KH550 and the B-OH in PBS, which reduced the agglomeration of the dispersed phase particles. It is well–established that the interparticle distance acts on the fracture behavior of blends. When the interparticle distance falls below the critical interparticle distance related to the matrix, the stress fields surrounding adjacent particles interact, leading to the ductile fracture of the blend.

**Figure 7:**
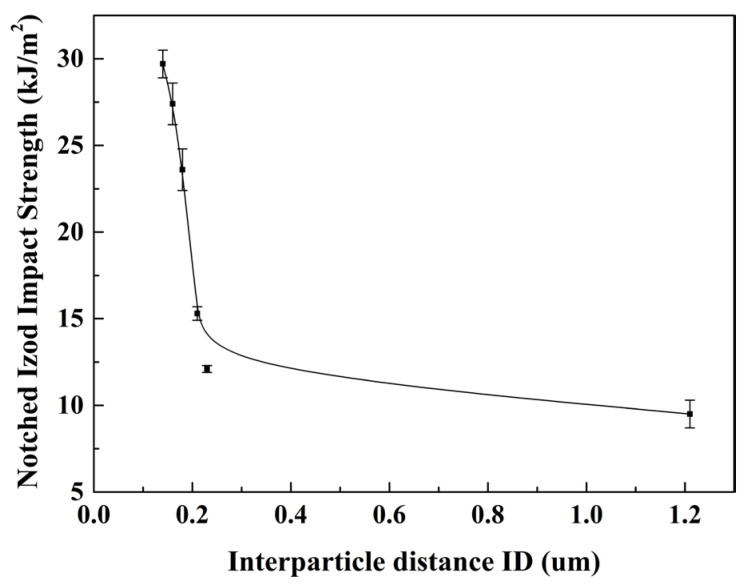
Notched Izod impact strength with interparticle distance *ID* for PA6/PBS blends with variation of KH550.

To further clarify the relationship between the interparticle distance in blends and impact toughness, the curve of *ID* versus notched Izod impact strength for PA6/PBS blends with varying contents of KH550 is depicted in Figure 7. It was observed that the interparticle distance exhibited a significant correlation with the notched Izod impact strength of the blends. In the absence of KH550 in the binary blend, the particles remained at a distance from each other. Consequently, the stress field within the matrix manifested as a mere superimposition of the stress fields surrounding isolated particles, leading to a brittle material behavior characterized by low-notched Izod impact strength. As the content of KH550 increased, the interparticle distance diminished. When the particles became sufficiently close, the stress field around one particle began to influence the stress fields of neighboring particles. In this scenario, the stress field no longer resembled a simple overlap; rather, it became an interaction of the stress fields surrounding adjacent particles, leading to a shear yield of the matrix [29,35]. This transition shifted the blend’s fracture behavior from brittle to ductile, significantly enhancing the notched Izod impact strength.

### 3.4. Mechanical Properties

**Figure 8:**
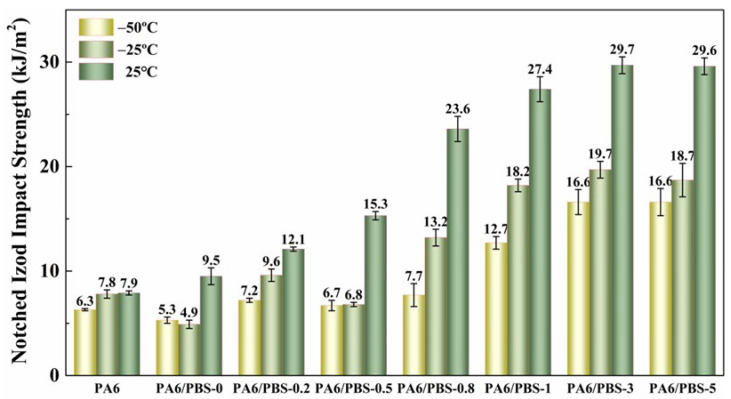
Notched Izod impact strength of neat PA6 and PA6/PBS blends at different temperatures.

The influence of the silane coupling agent on the notched Izod impact strength of the PA6/PBS blends was examined while maintaining a constant content of PBS across different temperatures, as shown in Figure 8. It was found that the notched Izod impact strength of the PA6/PBS blends at varying temperatures significantly improved with an increase in KH550. Specifically, the notched Izod impact strength improved from 7.9 kJ/m^2^ to 29.7 kJ/m^2^ at 25 °C and from 6.3 kJ/m^2^ to 16.6 kJ/m^2^ at −50 °C. This enhancement was attributed to the reduction in both the particle size and interparticle distance of PBS, as well as stronger interfacial adhesion of the two phases within blends. When the blends were subjected to impact, stress tended to concentrate in the dispersed phase. In blends with larger PBS particle sizes and weaker interfacial adhesion, this stress concentration phenomenon was intensified, making them more vulnerable to interfacial debonding under triaxial tensile stress, which subsequently resulted in lower notched Izod impact strength. Conversely, blends featuring smaller PBS particle sizes and closer interparticle distances combined with robust interfacial adhesion experienced a transformation from plane strain to plane stress upon impact. This interaction of the stress field around adjacent particles allowed the matrix to generate shear yielding, thereby enhancing the notched Izod impact strength.

**Figure 9:**
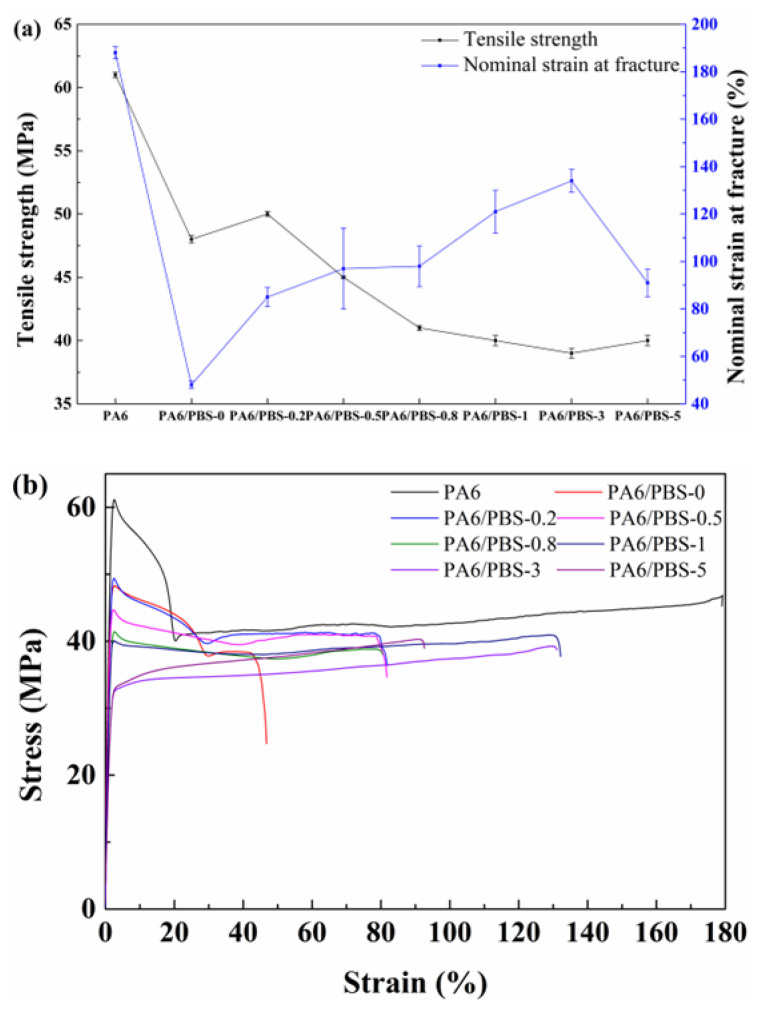
The tensile properties of neat PA6 and PA6/PBS blends: (**a**) tensile strength and nominal strain at fracture; (**b**) the curve of stress–strain.

The tensile strength and nominal strain at fracture of both neat PA6 and PA6/PBS blends are presented in Figure 9a. The introduction of PBS into PA6 resulted in a reduction in the tensile strength of the blends compared to that of pure PA6, primarily due to the lower modulus of PBS. In the absence of KH550, the binary blends exhibited weak adhesion at the two-phase interface, leading to the detachment of the PBS under uniaxial tensile stress. Consequently, both the tensile strength and nominal strain at fracture of the blends diminished. However, the incorporation of KH550 enhanced the interfacial adhesion between the two phases. During stretching, the tensile stress was effectively transferred from the matrix to the PBS phases, enabling the PBS phases to stretch alongside the matrix. Thus, the nominal strain at fracture of the blend increased with a higher content of KH550. Essentially, the PBS phases served a nailing function in the deformation of the matrix throughout the stretching process of the blends. It was important to note that the larger particle size of PBS intensified the nailing effect on matrix deformation, leading to a decrease in the tensile strength of the blend as the particle size of PBS was reduced. When the content of KH550 reached 5 wt%, the particle sizes of the PBS phases became sufficiently small, and the proximity of the particles resulted in significant interaction forces between them. This phenomenon gradually dominated the deformation mechanisms of the blends, ultimately causing an increase in their tensile strength and a reduction in the nominal strain at fracture once again.

Furthermore, neat PA6 exhibited double-yield behavior under uniaxial tensile deformation, as illustrated in Figure 9b. This phenomenon is characteristic of semi-crystalline polymers, with the prevailing explanation being the heterogeneous and homogeneous slips of crystal blocks [6]. At the initial stage of tensile deformation of PA6, the deformation occurs predominantly within the amorphous region. As the tensile strain increases, stress is gradually transferred from the amorphous region to the crystalline region, causing uniform shear of the crystals. Continued augmentation of tensile strain leads to the fragmentation of crystals into smaller blocks, resulting in mutual sliding between these crystal blocks. Consequently, the initial yield point is associated with the homogeneous shear of the crystals, while the subsequent yield point emerges from the sliding between smaller crystal blocks [36].

The incorporation of KH550 into the binary blend caused the PBS to react with KH550. This interaction increased the spacing between molecular chains and enhanced the available free volume for molecular chain movement, thereby facilitating uniform shear of crystals. Consequently, the first yield point gradually decreased as the content of KH550 rose. When the concentration of KH550 reached 3 wt%, residual unreacted KH550 acted as a lubricant between the molecular chains of the blends. Simultaneously, the small interparticle distance of PBS generated interaction forces that could lead to the formation of a partial interpenetrating polymer network of the PA6/PBS blends, resulting in the absence of a distinct yield point in the tensile curve. As the content of KH550 continued to rise to 5 wt%, the proximity of the PBS particles intensified the interaction forces between neighboring particles, which, in turn, led to an increase in the tensile strength.

Nylon is recognized for its high solubility parameter, approximately 13.6, which leads to poor interfacial compatibility when blended with other polymers. To enhance the compatibility of blends, graft treatment of elastomers is a common practice. However, this method can generate by-products or require excessive organic solvents, resulting in increased costs and complexity. In contrast, silane coupling agents offer a more straightforward and cost-effective solution. These agents form a “molecular bridge” between inorganic and organic materials, often improving the strength of polymers, although their contribution to toughness enhancement may be limited [37]. For instance, Shi et al. [38] demonstrated that silane-modified tetra-needle-shaped zinc oxide whiskers (T-ZnOw) can only increase the notch impact strength of PA6 by 31% at room temperature. We discovered that the amino group in KH550 can react with B-OH in PBS and with terminal carboxyl groups in PA6, making it an effective compatibilizer for PBS/PA6 blends. This approach not only streamlines the preparation process but also significantly enhances the impact strength of PA6 at room temperature by 276% and the notch strength at low temperatures by 163%. This advancement opens up new possibilities for the industrial application of PA6/PBS alloys.

## 4. Conclusions

In this work, PBS/PA6 binary blends were prepared via melt blending with KH550 as a compatibilizer. The influence of the silane coupling agent content on the interfacial adhesion of PA6/PBS blends was examined, along with the relationship between the interfacial adhesion of two phases and the mechanical properties of the blends. The results indicated that the amino group in KH550 reacted with the terminal carboxyl group in PA6 and the B-OH in PBS, thereby enhancing the interfacial adhesion between PBS and PA6. As the concentration of the KH550 increased, the improved interfacial adhesion among the blends resulted in a reduction in both the particle size and interparticle distance of the PBS phases. This change prompted a tough–brittle transition within the blend. The superior dispersion and robust interfacial adhesion of the blends improved the notched Izod impact toughness of PA6 from 7.9 kJ/m^2^ to 29.7 kJ/m^2^ at 25 °C and from 6.3 kJ/m^2^ to 16.6 kJ/m^2^ at −50 °C. Furthermore, a double-yield phenomenon was observed in the tensile curve of the blend, attributed to heterogeneous and homogeneous slips of crystal blocks. However, this phenomenon disappeared as the morphology of the blends transitioned from an initial “sea-island” structure to a partially interpenetrating polymer network with the addition of excessive KH550 within the blends.

## Figures and Tables

**Table 1:** Compositions of the prepared PA6/PBS blends.

Samples	PA6 (wt%)	PBS (wt%)	KH550 (wt%)	Antioxidant (wt%)
PA6/PBS-0	80	20	0	0.2
PA6/PBS-0.2	80	20	0.2	0.2
PA6/PBS-0.5	80	20	0.5	0.2
PA6/PBS-0.8	80	20	0.8	0.2
PA6/PBS-1	80	20	1	0.2
PA6/PBS-3	80	20	3	0.2
PA6/PBS-5	80	20	5	0.2

**Table 2:** Neat PA6 and PA6/PBS blends’ melting temperatures (T_m_), glass transition temperatures (T_g_), crystallization temperatures (T_c_), crystallization onset temperatures (T_c,onset_), melting enthalpies (Δ*H_m_*), crystallization enthalpies (Δ*H_c_*), and crystallinities (*χ_c_*).

Samples	T_m_ (°C)	Δ*H_m_* (J/g)	T_g_ (°C)	T_c_ (°C)	T_c,onset_ (°C)	Δ*H_c_* (J/g)	*χ_c_* (%)
PA6	223.3	52.57	52.4	176.3	195.2	58.63	22.9
PA6/PBS-0	222.1	32.51	53.2	191.4	196.2	43.39	17.7
PA6/PBS-0.2	222.4	37.03	53.5	190.8	196.2	47.02	20.2
PA6/PBS-0.5	222.4	34.88	49.2	190.4	195.2	44.82	19.1
PA6/PBS-0.8	221.9	33.63	51.9	189.3	195.2	44.46	18.4
PA6/PBS-1	222.8	35.33	51.4	190.4	195.2	44.41	19.5
PA6/PBS-3	222.8	36.19	52.8	188.4	193.2	44.84	20.3
PA6/PBS-5	222.2	31.33	46	187.7	193.2	41.91	17.9

**Table 3:** The *d_w_* and *ID* values of PA6/PBS blends with different KH550 contents.

KH550 (Mass Fraction)	0 wt%	0.2 wt%	0.5 wt%	0.8 wt%	1 wt%	3 wt%
*d_w_* (μm)	3.63	0.68	0.62	0.52	0.47	0.39
*ID* (μm)	1.21	0.23	0.21	0.18	0.16	0.14

## Data Availability

The original contributions presented in this study are included in the article. Further inquiries can be directed to the corresponding author.
